# Pyogenic Hepatic Abscess: Changing Therapy?

**DOI:** 10.1155/1998/83967

**Published:** 1998-08

**Authors:** John W. C. Chen, James Toouli

**Affiliations:** Gastrointestinal Surgical Unit Department of Surgery Flinders Medical Centre Bedford Park 5042 South Africa


### Pyogenic Hepatic Abscess: Changing Therapy?

### ABSTRACT

*Huang, C-J., Pitt, H. A., Lipsett, P. A., Osterman Jr., F. A., Lillemoe, K. D., Cameron, J. L. and Zuiderma, G. D. (1996) Pyogenic Hepatic Abscess: Changing trends over 42 years. Annals of surgery; 223: 600-609.*

***Objective*: The authors document changes in the etiology, diagnosis, bacteriology, treatment, and outcome of patients with pyogenic hepatic abscesses over the past 4 decades.**
***Summary Background Data*: Pyogenic hepatic abscess is a highly lethal problem. Over the past 2 decades, new roentgenographic methods, such as ultrasound, computed tomographic scanning, direct cholangiography, guided aspiration, and percutaneous drainage, have altered both the diagnosis and treatment of these patients. A more aggressive approach to the management of hepatobiliary and pancreatic neoplasms also has resulted in an increased incidence of this problem.**
***Methods*: The records of 233 patients with pyogenic liver abscesses managed over a 42-year period were reviewed. Patients treated from 1952 to 1972 (n = 80) were compared with those seen from 1973 to 1993 (n = 153).**
***Results*: From 1973 to 1993, the incidence increased from 13 to 20 per 100,000 hospital admissions ($p<0.01$). Patients managed from 1973 to 1993 were more likely ($p<0.01$) to have an underlying malignancy (52% vs. 28%) with most of these (81%) being a hepatobiliary or pancreatic cancer. The 1973 to 1993 patients were more likely ($p< .05$) to be infected with streptococcal (53% vs. 30%) or *Pseudomonas* (30% vs. 9%) species or to have mixed bacterial and fungal (26% vs. 1%) infections. The recent patients also were more likely ($p<0.05$) to be managed by percutaneous abscess drainage (45% vs. 0%). Despite having more underlying problems, overall mortality decreased significantly ($p<0.01$) from 65% (in 1952 to 1972 period) to 31% (in 1973 to 1993 period). This reduction was greatest for patients with multiple abscesses (88% vs. 44%; $p<0.05$) with either a malignant or a benign biliary etiology (90% vs. 38%; $p<0.05$). Mortality was increased ($p<0.02$) in patients with mixed bacterial and fungal abscesses (50%). From 1973 to 1993, mortality was lower ($p=0.19$) with open surgical as opposed to percutaneous abscess drainage (14% vs. 26%).**
***Conclusions*: Significant changes have occurred in the etiology, diagnosis, bacteriology, treatment, and outcome of patients with pyogenic hepatic abscesses over the past 4 decades. However, mortality remains high, and proper management continues to be a challenge. Appropriate systemic antibiotics and fungal agents as well as adequate surgical, percutaneous, or biliary drainage are required for the best results.**

*Keywords:* Liver abscess, hepatic abscess, pyogenic liver abscess

### PAPER DISCUSSION

Several series on pyogenic liver abscess (PLA) have been published over the years since the classic report by Ochsner and De Bakey in 1938 [1]. All have been retrospective reviews. These have given considerable insight into the disease pattern and progress made on the diagnostic as well as therapeutic front. Recent series have evaluated risk factors, prognostic factors, and modern therapeutic strategies. Despite significant progress with early diagnosis, improved antibiotics and intensive supportive treatment, pyogenic liver abscess still carries a significant mortality.

Pyogenic liver abscess remains a rare disease. Most published series in major institutions averaged around 2 to 8 cases a year [2,3,4,5,6,7,8,9,10]. Due to the relative rarity of the condition, there has not been any prospective randomised controlled trial looking at non-operative treatment versus open surgical drainage. A number of small series have shown successful outcome using antibiotics alone or antibiotics plus aspiration [11,12,13]. In other series, antibiotics plus percutaneous drainage has been associated with good results [2,3,4,7]. However, in some series an unfavourable outcome has been reported using these non-operative measures [5,6] All of these series have reported experiences with a small number of patients.

Recently, a relative large retrospective review of the treatment of pyogenic liver abscess at a major centre has been reported. Huang *et al.* [14] have reported their experience over a period of forty-two years at John Hopkins Hospital. They reviewed a current series of 153 patients over a period of 21 years (1973 – 1993) and compared it with the experience of a previously published series of 80 patients (1952 – 1972) [15].

They noted an increase in incidence of PLA during the second period (13 to 20 per 100,000 hospital admissions) and this was attributed to an increase in the number of patients with underlying malignancy (28% to 52%), and an associated increase in the use of biliary stents for the treatment of inoperable cancer. During the same period, there was a significant reduction in mortality (65% to 31%) which was attributed to improvement in diagnosis and development of percutaneous and non-operative biliary drainage techniques. This improvement in mortality is consistent with other recently published series [4, 5, 6, 7].

Liver malignancy and malignant biliary obstruction are the major causes of PLA as in other series [2, 5, 6]. Of interest are the patients who developed PLA after hepatic artery catheterisation for treatment of liver secondaries. This complication had been previously reported [16], and as in this series, it occurs despite use of prophylactic antibiotics. The bacteriology of the pyogenic abscess from nearly all series indicated that the most likely organisms are gram negative rods, and most likely to have an enteric origin. Hence, these patients may be predisposed to PLA as a result of either decreased immunity or the necrosed tumour becomes the perfect medium for enteric bacteria to seed, probably via the portal venous system.

The bacteriology of the abscesses in this series is similar to other major series, where enteric organisms predominate. E Coli and Klebsiella are by far the most common, followed by streptococcus. In this series, the incidence of streptococcus is greater as compared to the first period. In addition, there was a high incidence of resistant organisms present, probably due to frequent use of antibiotics during stent manipulation. A dramatic change has been the occurrence of fungi in 26% of abscess cultures in the second series compared to 1% in the first series and reflects the wide and frequent use of broad spectrum antibiotics, particularly in patients with stents who have frequent changes of the stents and intermittent episodes of cholangitis.

High quality ultrasonography and CT scanning have contributed to the greater accuracy of diagnosis between the two periods reviewed. However, cholangiography still remains a useful investigation in up to 45% of patients. MRI has not been shown to be useful, but it has not been adequately evaluated.

The trend towards increasing use of percutaneous drainage of PLA and systemic antibiotics is noted in recent series [2, 3, 4, 7, 8, 11, 13, 17]. All series adopted a selective approach with percutaneous aspiration or drainage coupled with systemic antibiotics for selected cases of PLA. In the John Hopkin's series, percutaneous drainage was not used in the first period. However, 45% of the patients in the second period were treated with percutaneous drainage. The overall mortality has decreased significantly from 65% to 31% and this is attributed to better diagnosis, improved antibiotics and the use of percutaneous approach. However, a comparison of the mortality of patients having the percutaneous approach (26%) versus open surgery (14%) shows a trend favouring open surgery. The authors noted that this apparent difference was due to patient selection and that the higher mortality in those patients having percutaneous treatment was attributed to the underlying disease (often a high cholangiocarcinoma) and reflected the end stages of the disease.

The use of antibiotics alone in this series was associated with 100% mortality reinforcing the view that PLA, as with all abscesses, requires drainage in addition to the use of appropriate antibiotics.

Univariate analysis of risk factors was performed. Risk factors found to be significant are comparable to other series [3, 8, 18, 19]. Risk increases in the presence of multiple abscesses, associated malignancy, jaundice, hypoalbuminaemia, leukocytosis, bilirubinaemia and the presence of fungus infection.

This large series of patients with PLA treated at a single major institution provides important insight into the changing aetiology of this disease. It has illustrated that it still remains as a disease with high mortality, but often this mortality reflects the end stage of the underlying pathology. The trend away from open surgical approaches is well illustrated, but the series also shows that drainage needs to be achieved in addition to the use of appropriate antibiotics. There is a cautionary note regarding the use of broad spectrum antibiotics in patient with indwelling stents, as this practice has generated fungal contamination of liver abscesses with associated increased mortality. The drainage of pyogenic abscess may be achieved via a percutaneous approach in most patients, but in addition, biliary drainage and a combination of laparoscopic plus percutaneous drainage may have a place. In a selected group of patients, resection of an infected segment of liver may be the best and most conservative form of treatment.

### *References*

[1] Ochsner, A., DeBakey, M. and Murray, S. (1938). Pyogenic abscess of the liver. *American Journal of Surgery*, **40**, 292–319.

[2] Rintoul, R., O'Riordain, M. G., Laurenson, I. F., Crosbie, J. L., Allan, P. I. and Garden, O. J. (1996). Changing management of pyogenic liver abscess *British Journal of Surgery*, **83**, 1215–1218.

[3] Chu, K. M., Fan, S. T., Lai, E. C. Lo, C. M. and Wong, J. (1996). An audit of experience over the past decade. *Archive of surgery*, **131**(2), 148–52.

[4] Stain, S. C., Yellin, A. E., Donovan, A. J. and Brien, H. W. (1991). Pyogenic liver abscess. Modern treatment. *Archive of Surgery*, **126**(8), 991–6

[5] Branum, G. D., Tyson, G. S., Branum, M. A. and Meyers, W. C. (1990). Hepatic abscess: Changes in etiology, diagnosis, and management. *Annals of Surgery*, **212**, 655–662.

[6] Cohen, J. L., Martin, F. M., Rossi, R. L., Schoetz, D. J. Jr., Liver abscess. (1989). The need for complete gastrointestinal evaluation. *Archive of Surgery*, **124**(5), 561–4.

[7] Farges, O., Leese, T. and Bismuth, H. (1988). Pyogenic liver abscess: an improvement in prognosis. *British Journal of Surgery*, **75**(9), 862–5.

[8] Greenstein, A. J., Lowenthal, D., Hammer, G. S., Schaner, F. and Aufses, A. H. Jr, (1984). Continuing changing patterns of disease in pyogenic liver abscess: a study of 38 patients. *American Journal of Gastroenterology*, **79**(3), 217–26.

[9] Northover, J. M., Jones, B. J. Dawson, J. L. and Williams, R. (1982). Diculties in the diagnosis and management of pyogenic liver abscess. *British Journal of Surgery*, **69**(1), 48–51.

[10] Lazarchick, J., De Souza e Silva, N., Nichols, D. R. and Washington, J. A. (1973). Pyogenic Liver Abscess. *Mayo Clinic Proceedings*, **48**, 349–355.

[11] Berger, L. A. and Osbourne, D. R. (1982). Treatment of pyogenic liver abscesses by percutaneous needle aspiration. *Lancet*, **16**, 132–134.

[12] Herbert, D. A., Rothman, J., Simmons, F., Fogel, D. A., Wilson, S. and Ruskin, J. (1982). Pyogenic liver abscesses: Successful non-surgical therapy. *Lancet*, **16**, 134–136.

[13] Giorgio, A., Tarantino, L., Mariniello, N., Francica, G., Scala, E., Amoroso, P., Nuzzo, A. and Rizzatto, G. (1995). Pyogenic liver abscesses: 13 years of experience in percutaneous needle aspiration with US guidance. *Radiology*, **195**(1), 122–4.

[14] Huang, C. J., Pitt, H. A., Lipsett, P. A., Osterman, F. A., Lillemoe, K. D., Cameron, J. L. and Zuidema, G. D. (1996). Pyogenic hepatic abscess: Changing trends over 42 years. *Annals of Surgery*, **223**, 600–609.

[15] Pitt, H. A. and Zuidema, G. D. (1975). Factors influencing mortality in the treatment of pyogenic hepatic abscess. *Surgery Gynaecology and Obsteric*, **140**, 228–234.

[16] Wong, E., Khardori, N., Carrgsco, C. H., Wallace, S., Patt, Y. and Bobey, G. P. (1991). Infectious complications of hepatic artery catherisation procedures in patients with cancer. *Review of Infectious Disease*, **13**, 583–589.

[17] McDonald, M. I., Corey, G. R., Gallis, H. A. and Durack, D. T. (1984). Single and multiple pyogenic liver abscesses. Natural history, diagnosis and treatment, with emphasis on percutaneous drainage. *Medicine-Baltimore*, **63**(5), 291–302.

[18] Chou, F. F., Sheen-chen, S. M., Chen, Y. S. and Lee, T. Y. (1995). The comparison of clinical course and results of treatment between gas-forming and non-gas-forming pyogenic liver abscess. *Archive of Surgery*, **130**(4), 401–5; discussion 406.

[19] Lee, K. T., Sheen, P. C., Chen, J. S. and Ker, C. G. (1991). Pyogenic liver abscess: Multivariate analysis of risk factors. *World Journal of Surgery*, **15**, 372–377.

John W. C. Chen and
James Toouli
Gastrointestinal Surgical Unit
Department of Surgery
Flinders Medical Centre
Bedford Park
South Australia 5042

